# Successful Management of a Large Pediatric Traumatic Pneumothorax Using a Thoracic Vent Device After Blunt Chest Injury: A Case Report

**DOI:** 10.7759/cureus.111950

**Published:** 2026-07-02

**Authors:** Jayanjali Bodavula, Melika Babadi, Morgan W Stewart, Brett Dunbar

**Affiliations:** 1 Osteopathic Medicine, Kansas City University, Joplin, USA; 2 Osteopathic Medicine, Kansas City University, Kansas City, USA; 3 General Surgery, Mercy Hospital Pittsburg, Pittsburg, USA

**Keywords:** chest trauma, minimally invasive drainage, pediatric traumatic pneumothorax, small-bore pleural catheter, thoracic vent device, uresil thora-vent

## Abstract

Traumatic pneumothorax is a common complication of blunt thoracic trauma and is traditionally managed with tube thoracostomy, particularly in larger or symptomatic cases requiring pleural decompression. Thoracic vent devices, such as the UreSil Thora-Vent, represent a less invasive pleural drainage option for the management of spontaneous or iatrogenic pneumothorax. However, literature regarding their use in traumatic pneumothorax, particularly in pediatric patients, remains limited. We present a case of a 13-year-old girl who developed a large left-sided traumatic pneumothorax after blunt thoracic trauma sustained during a softball collision. Computed tomography demonstrated a large left-sided pneumothorax and a nondisplaced left sixth rib fracture. Despite the extent of injury, the patient remained hemodynamically stable and maintained adequate oxygenation on room air. A thoracic vent device was placed in the emergency department under ketamine sedation, resulting in immediate air return and near-complete radiographic lung re-expansion. During hospitalization, the thoracic vent device was initially managed on water seal, and serial imaging demonstrated improvement in lung re-expansion. However, subsequent imaging revealed a transient interval increase in pneumothorax size, prompting transition to continuous suction. Despite these fluctuating radiographic findings, the patient remained clinically stable without worsening respiratory distress or need for supplemental oxygen. The thoracic vent device was removed on hospital day 3, and the patient was discharged in stable condition on hospital day 4. Follow-up chest radiography one week later demonstrated complete resolution of the pneumothorax. This case demonstrates the feasibility of thoracic vent use as a less invasive management strategy in carefully selected, hemodynamically stable pediatric patients with trauma. It also highlights the importance of close serial imaging, ongoing clinical reassessment, and a clearly defined escalation strategy when employing less invasive pleural drainage approaches in pediatric traumatic pneumothorax management.

## Introduction

Traumatic pneumothorax is a known sequela of traumatic or blunt thoracic injury and is traditionally managed with tube thoracostomy [1]. Management strategies for pediatric pneumothorax are largely extrapolated from adult guidelines because pediatric-specific data remain limited [1]. While effective, conventional chest tube placement is invasive and may be associated with patient discomfort, restricted mobility, prolonged hospitalization, and procedural complications [1,2].

In recent years, interest has increased in less invasive pleural drainage strategies, particularly with the growing use of small-bore drainage systems in select pneumothorax populations [3]. Thoracic vent devices, such as the UreSil Thora-Vent, are small-bore pleural drainage systems that allow continuous evacuation of pleural air through a one-way valve mechanism [2]. Their use has been described primarily in spontaneous or iatrogenic pneumothorax in adult populations [2]. However, published literature regarding thoracic vent device use in traumatic pneumothoraces, particularly in pediatric patients with trauma, is minimal [4,5]. As a result, the role of these devices in traumatic pediatric pneumothorax remains poorly defined. 

We present the case of a 13-year-old girl with a large traumatic pneumothorax following blunt thoracic injury who was successfully managed with a thoracic vent device without escalation to conventional tube thoracostomy. This case highlights the potential feasibility of thoracic vent use in select hemodynamically stable pediatric patients with trauma, and underscores the need for further investigation into their role in pediatric traumatic pneumothorax management.

## Case presentation

A 13-year-old girl with no significant past medical history presented to the emergency department (ED) after sustaining blunt thoracic trauma during a softball game. Earlier that day, she had jumped off first base to catch a ball when another player, wearing a helmet, collided headfirst into her left chest. She was airborne at the time of impact and subsequently landed on her left chest. She denied any head trauma or loss of consciousness. Although she was initially able to continue playing for two additional games, she later developed progressively worsening left-sided chest pain and shortness of breath over the course of several hours, prompting presentation to the ED. On presentation, the patient appeared extremely anxious and in acute distress. Vital signs were notable for tachycardia with a heart rate of 104 beats per minute and tachypnea with a respiratory rate of 44 breaths per minute. Blood pressure was 134/82 mmHg, and oxygen saturation was 99% on room air, without the need for supplemental oxygen. Physical examination demonstrated decreased air movement and breath sounds throughout the left lung fields, along with significant tenderness and swelling over the left chest wall. The remainder of the examination was unremarkable.

Computed tomography (CT) of the chest revealed a large left-sided pneumothorax and a nondisplaced anterolateral fracture of the left sixth rib (Figure 1). No intra-abdominal injuries were identified. An 11-French UreSil Thora-Vent device (thoracic vent device; UreSil, LLC, Skokie, Illinois, USA) was placed in the ED under ketamine sedation. The device was inserted at approximately the left fourth intercostal space along the midclavicular line, and immediate return of air was noted upon entry into the pleural space. It was then secured and connected to continuous wall suction at -20 cm H_2_0. A post-procedural chest radiograph obtained two hours later demonstrated near-complete re-expansion of the left lung with a small residual apical pneumothorax and appropriate positioning of the device (Figure 2).

**Figure 1:**
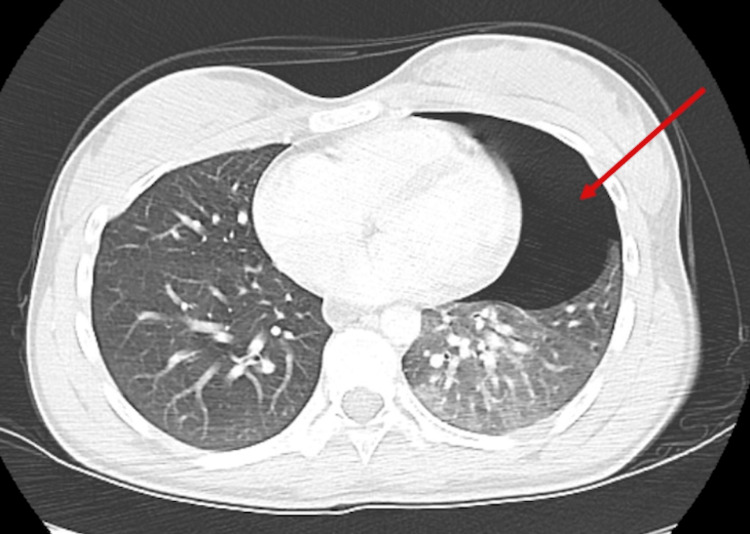
Computed tomography of the chest demonstrating a large left-sided pneumothorax (arrow) following blunt thoracic trauma

**Figure 2:**
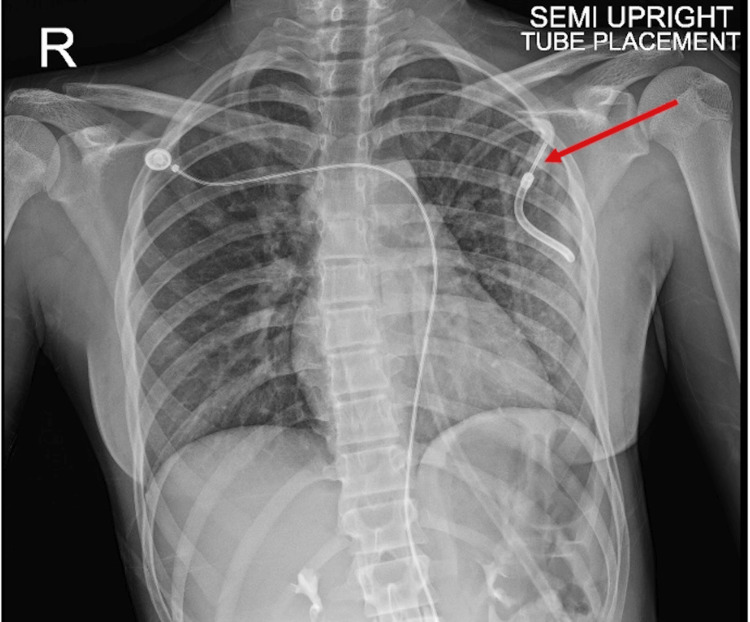
Post-procedural chest radiograph obtained two hours after thoracic vent placement demonstrating near-complete re-expansion of the left lung with appropriate positioning of the device (arrow)

The patient was subsequently transferred to a tertiary care center for further management. On arrival, she remained hemodynamically stable and in no respiratory distress, maintaining adequate oxygenation on room air, with the thoracic vent device in place.

During her hospital course, the thoracic vent device was initially maintained on water seal, with serial chest radiographs demonstrating improvement in lung expansion. However, subsequent imaging revealed a mild interval increase in the size of the pneumothorax, prompting transition to continuous wall suction at -20 cm H_2_O. Despite these fluctuating radiographic changes, the patient remained clinically stable without increased work of breathing.

On hospital day 3, the thoracic vent device was removed, at which time a small residual left-sided pneumothorax remained. Following removal, repeat imaging demonstrated no progression or enlargement of the pneumothorax. The patient was discharged on hospital day 4 in stable condition. A follow-up chest radiograph obtained one week later demonstrated complete resolution of the pneumothorax (Figure 3). No device-related complications requiring intervention, supplemental oxygen, or escalation to conventional tube thoracostomy occurred during hospitalization or at 1-week follow-up. A detailed timeline of the patient’s clinical course is summarized in Table 1.

**Figure 3:**
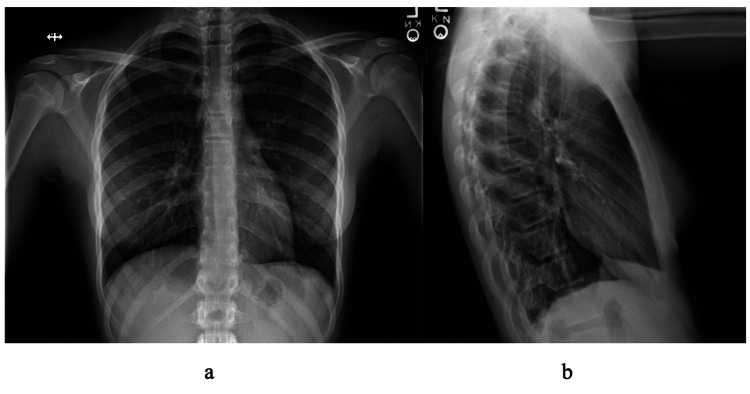
Follow-up chest radiographs obtained 1 week after discharge demonstrating complete resolution of the left-sided pneumothorax. (a) Posteroanterior view. (b) Lateral view

**Table 1:** Clinical timeline of injury, intervention, and hospital course ED: emergency department; CT: computed tomography

Day	Clinical events
Day 0 (injury)	Player-to-player collision during a softball game resulting in blunt left-sided thoracic trauma.
Day 0 (ED presentation)	Presented with chest pain and shortness of breath. CT chest demonstrated a large left-sided pneumothorax and left 6^th^ rib fracture. Thoracic vent device placed with near-complete re-expansion of the left lung.
Day 1 (transfer)	Transferred to a tertiary care center with a thoracic vent device in place. Remained hemodynamically stable without respiratory distress.
Hospital day 1-2	Initially managed on water seal with improvement in lung expansion. Subsequent imaging showed a mild interval increase in pneumothorax, prompting transition to continuous wall suction.
Hospital day 3	The thoracic vent device was removed with a small residual pneumothorax present.
Hospital day 4	Discharged in stable condition.
One-week follow-up	Chest radiograph demonstrated complete resolution of pneumothorax.

## Discussion

Historically, tube thoracostomy with a large-bore chest drain has been the standard intervention for traumatic pneumothorax [6]. However, tube thoracostomy carries significant procedural risks, including malposition, insertion-site infection, neurovascular injury, and recurrent pneumothorax, all of which contribute to patient morbidity and healthcare costs [6,7]. A recent meta-analysis of randomized controlled trials comparing small-bore devices (≤14 French) to large-bore chest tubes (≥28 French) in adult trauma patients found that small-caliber tubes offer similar treatment efficacy with the additional advantages of easier bedside placement, less procedural trauma, reduced insertion-site pain, and lower wound complication rates [8]. These findings have contributed to a trend toward smaller drainage systems and, for select stable patients, observation as an alternative to reflexive large-bore tube placement [8].

A fundamental challenge in the pediatric setting is that pneumothorax management guidelines from the American College of Chest Physicians, European Respiratory Society, and British Thoracic Society are specific to adult patients and have not been formally addressed in pediatric populations, resulting in significant management variation [1]. Children sustain thoracic injuries differently than adults because the pediatric chest wall is more compliant, allowing kinetic energy to be transmitted to intrathoracic structures without producing obvious rib fractures [9]. As a result, pulmonary contusions are often more common than radiographically apparent rib fractures in pediatric patients with trauma [9]. This anatomical distinction complicates the direct application of adult-derived management thresholds to children, and pediatric-specific evidence for traumatic pneumothorax management remains limited [1,9].

The UreSil Thora-Vent is a small-bore pleural drainage system consisting of a flexible polyurethane catheter connected to a one-way valve housing. Although the device may be managed on a water seal in hospitalized patients, it is also designed to permit ambulatory pleural decompression without requiring connection to a traditional underwater seal device [2]. In a prospective pilot study of 18 patients with spontaneous or iatrogenic pneumothorax, technical success was achieved in all cases, and complete lung re-expansion within 24 hours was documented in 88.9% of patients, with a mean procedural pain score of 2.4 out of 10 during daily activity and no major complications [2]. Despite these favorable characteristics, published experience with thoracic vent devices in pediatric traumatic pneumothorax is virtually absent, and the existing evidence base derives almost exclusively from non-traumatic populations in adults [2].

The present case highlights the potential feasibility of ambulatory small-bore drainage in a carefully selected pediatric patient. Our patient presented with a large, CT-confirmed left-sided traumatic pneumothorax accompanied by an ipsilateral rib fracture, findings that would conventionally prompt large-bore tube thoracostomy [6,10]. Instead, a thoracic vent device placed in the ED under procedural sedation achieved immediate air return and near-complete lung re-expansion within 2 hours of placement. Although subsequent imaging revealed a transient increase in pneumothorax size during initial water-seal management, the patient remained hemodynamically stable and never required supplemental oxygen or escalation to conventional tube thoracostomy. Currently, there are no pediatric-specific guidelines defining thresholds for transitioning from water-seal management to continuous suction. Therefore, management should be individualized based on serial clinical assessment and imaging. Accordingly, in our patient, an interval increase in pneumothorax size despite continued clinical stability prompted transition to continuous wall suction at -20 cm H_2_O, resulting in subsequent lung re-expansion without the need for conventional tube thoracostomy. Complete radiographic resolution was confirmed at one-week follow-up, with a total hospital stay of four days.

These findings support the growing recognition that not all traumatic pneumothoraces in children require immediate large-bore tube thoracostomy, and management should instead be guided by hemodynamic stability, pneumothorax size, and clinical symptoms [6,11]. In the absence of validated pediatric-specific guidelines, clinicians must individualize management using adult evidence and patient-specific factors, selecting tube size and procedural invasiveness according to clinical need rather than reflexively escalating intervention [1]. Our patient's uneventful clinical course without escalation to large-bore drainage illustrates that this stepwise, less invasive approach to intervention may be feasible in appropriately selected pediatric patients with trauma.

Several clinical features of this case may help inform future patient selection for thoracic vent use in pediatric traumatic pneumothorax. Hemodynamic stability, adequate oxygenation on room air, absence of hemothorax, and no requirement for positive-pressure ventilation were the key factors that made our patient a candidate for a less invasive approach [6,12]. Although the patient demonstrated transient interval worsening of the pneumothorax following initial improvement under water seal management, she remained clinically stable and ultimately achieved complete resolution without requiring escalation to conventional tube thoracostomy. This case suggests that thoracic vent use may be feasible in carefully selected pediatric trauma patients when combined with close serial imaging and a clearly defined escalation strategy. Similar findings have been described by Banks et al., who noted that a subset of carefully selected stable patients ultimately required delayed tube thoracostomy following clinical deterioration [13]. Accordingly, clinicians should maintain a low threshold for escalation if clinical or radiographic worsening occurs.

This case report has important limitations. Single cases cannot establish generalizability, and the favorable outcome observed does not constitute evidence of equivalence or superiority of thoracic vent devices over tube thoracostomy in pediatric traumatic pneumothorax. The optimal patient selection criteria, maximum amenable pneumothorax size, and standardized escalation thresholds for this approach remain undefined in the pediatric trauma context, and prospective comparative studies are needed before evidence-based recommendations can be established.

## Conclusions

This case demonstrates the successful management of a large traumatic pneumothorax in a hemodynamically stable pediatric patient using a thoracic vent device without escalation to conventional tube thoracostomy. Despite a fluctuating radiographic course requiring transition from water seal to continuous suction, the patient remained clinically stable and ultimately achieved complete resolution of the pneumothorax. Thoracic vent devices, therefore, may represent a feasible, less invasive management option in carefully selected pediatric patients with trauma, particularly those who remain hemodynamically stable without significant respiratory compromise or the need for positive-pressure ventilation. However, careful patient selection, close serial imaging, ongoing clinical reassessment, and a low threshold for escalation to conventional tube thoracostomy remain essential. Further studies are needed to better define patient selection criteria, optimal management protocols, and clinical outcomes associated with thoracic vent device use in pediatric traumatic pneumothorax.
